# Direct Binding of Retromer to Human Papillomavirus Type 16 Minor Capsid Protein L2 Mediates Endosome Exit during Viral Infection

**DOI:** 10.1371/journal.ppat.1004699

**Published:** 2015-02-18

**Authors:** Andreea Popa, Wei Zhang, Megan S. Harrison, Kylia Goodner, Teymur Kazakov, Edward C. Goodwin, Alex Lipovsky, Christopher G. Burd, Daniel DiMaio

**Affiliations:** 1 Department of Genetics, Yale School of Medicine, New Haven, Connecticut, United States of America; 2 Department of Cell Biology, Yale School of Medicine, New Haven, Connecticut, United States of America; 3 Department of Therapeutic Radiology, Yale School of Medicine, New Haven, Connecticut, United States of America; 4 Department of Molecular Biophysics & Biochemistry, Yale School of Medicine, New Haven, Connecticut, United States of America; 5 Yale Cancer Center, New Haven, Connecticut, United States of America; National Institute of Allergy and Infectious Diseases, National Institutes of Health, UNITED STATES

## Abstract

Trafficking of human papillomaviruses to the Golgi apparatus during virus entry requires retromer, an endosomal coat protein complex that mediates the vesicular transport of cellular transmembrane proteins from the endosome to the Golgi apparatus or the plasma membrane. Here we show that the HPV16 L2 minor capsid protein is a retromer cargo, even though L2 is not a transmembrane protein. We show that direct binding of retromer to a conserved sequence in the carboxy-terminus of L2 is required for exit of L2 from the early endosome and delivery to the *trans*-Golgi network during virus entry. This binding site is different from known retromer binding motifs and can be replaced by a sorting signal from a cellular retromer cargo. Thus, HPV16 is an unconventional particulate retromer cargo, and retromer binding initiates retrograde transport of viral components from the endosome to the *trans*-Golgi network during virus entry. We propose that the carboxy-terminal segment of L2 protein protrudes through the endosomal membrane and is accessed by retromer in the cytoplasm.

## Author Summary

The human papillomaviruses are important carcinogens, but little is known about how these non-enveloped viruses traffic to the nucleus, the site of genome replication. We use imaging, biochemical, and genetic techniques to show that a multi-subunit intracellular trafficking machine known as retromer binds directly to the L2 minor capsid protein in the virus particle to initiate its transport from the endosome to other membrane-bound organelles farther inside the cell. Most notably, knock-down of retromer expression or mutation of newly identified retromer binding sites in L2 cause the accumulation of incoming HPV16 capsids in the endosome and prevent trafficking to the Golgi. These defects can be corrected by insertion of a retromer binding site from a cellular cargo. Because all previously known retromer cargoes are cellular transmembrane proteins, the virus represents a new class of retromer cargo. In addition to elucidating the mechanism of viral endosome escape, these results suggest that retromer may play a more versatile role in cell biology than previously recognized.

## Introduction

The human papillomaviruses are small non-enveloped viruses responsible for approximately 5% of human cancer deaths worldwide [1]. The cellular mechanisms involved in HPV infection are poorly understood, but they represent potential sites of anti-viral intervention. HPV consists of an ~8000 base-pair double-stranded DNA viral genome packaged in the viral capsid, which is composed of 360 molecules of the major capsid protein, L1, and up to 72 molecules of the minor capsid protein, L2, which is largely buried inside the L1 shell [2–4]. Initial binding of virus to cells is mediated by the interaction between the L1 capsid protein and heparan sulfate proteoglycans [5–8]. After cell binding, L1 and L2 undergo conformational changes, which allow cleavage of the amino-terminus of L2 at the cell surface by the protease furin [9–12]. HPV is then transferred to an as-yet-unidentified cell-surface receptor and internalized [13–17]. Disassembly of the capsid is initiated by acidification of the endosomal lumen by the vacuolar ATPase, and L1, L2, and viral DNA then traffic via retrograde pathways to the Golgi apparatus and endoplasmic reticulum [5,16–24]. Cell cycle progression and nuclear envelope breakdown appear required for HPV entry into the nucleus, where viral gene expression and DNA replication occur [25,26]. During virus trafficking, the L1 protein dissociates from the viral DNA, but the 473-amino acid L2 protein is required for efficient trafficking of the viral genome to the nucleus and remains associated with the genome during nuclear entry [20,27–34].

We performed a genome-wide siRNA screen to identify host cell genes required for HPV16 infection and discovered that infection of HeLa cervical cancer cells and immortalized cervical keratinocytes requires retromer, a cytoplasmic endosomal coat complex that mediates export of cellular transmembrane proteins from the endosome to the trans-Golgi network (TGN) or plasma membrane [22,35,36]. The cargo recognition core of the retromer consists of three subunits, Vps26, Vps29, and Vps35, all of which are required for retromer-mediated endosomal sorting and for HPV infection [22]. In cells depleted of retromer, HPV components fail to arrive at the TGN [22]. In addition, retromer is present in a stable complex with viral capsid proteins in infected cells [22].

The mechanism by which retromer supports HPV trafficking is unknown. All known retromer cargos are cellular, integral membrane proteins [35,36]. Retromer recognizes sorting signals located in the cytoplasmic domain of these transmembrane protein cargos at the endosomal membrane to effect packaging of the cargo into budding vesicles or tubules that later fuse with target membranes to deliver the cargo to its destination. Unlike known retromer cargos, the non-enveloped HPV capsid is particulate and lacks transmembrane proteins. Furthermore, when the capsid is in the endosomal lumen during the early stages of infection, it is separated from retromer in the cytoplasm by the endosomal membrane. It is possible that retromer acts indirectly by mediating trafficking of a cellular transmembrane protein that is essential for some step in HPV entry. Alternatively, HPV might have developed a strategy to access retromer even when the capsid itself is in the lumen of the endosome. Finally, virus might exit from the endosome into the cytoplasm where it can be recognized by retromer, which later mediates its entry into the Golgi. The experiments reported here demonstrate that retromer directly binds to the minor capsid protein of incoming HPV and that this interaction mediates export of virus particles from the early endosome into the retrograde vesicular pathway during the early stages of intracellular trafficking of incoming HPV.

## Results

### Non-canonical retromer sorting motifs in the carboxy-terminus of L2 are required for infectivity

**Fig 1:**
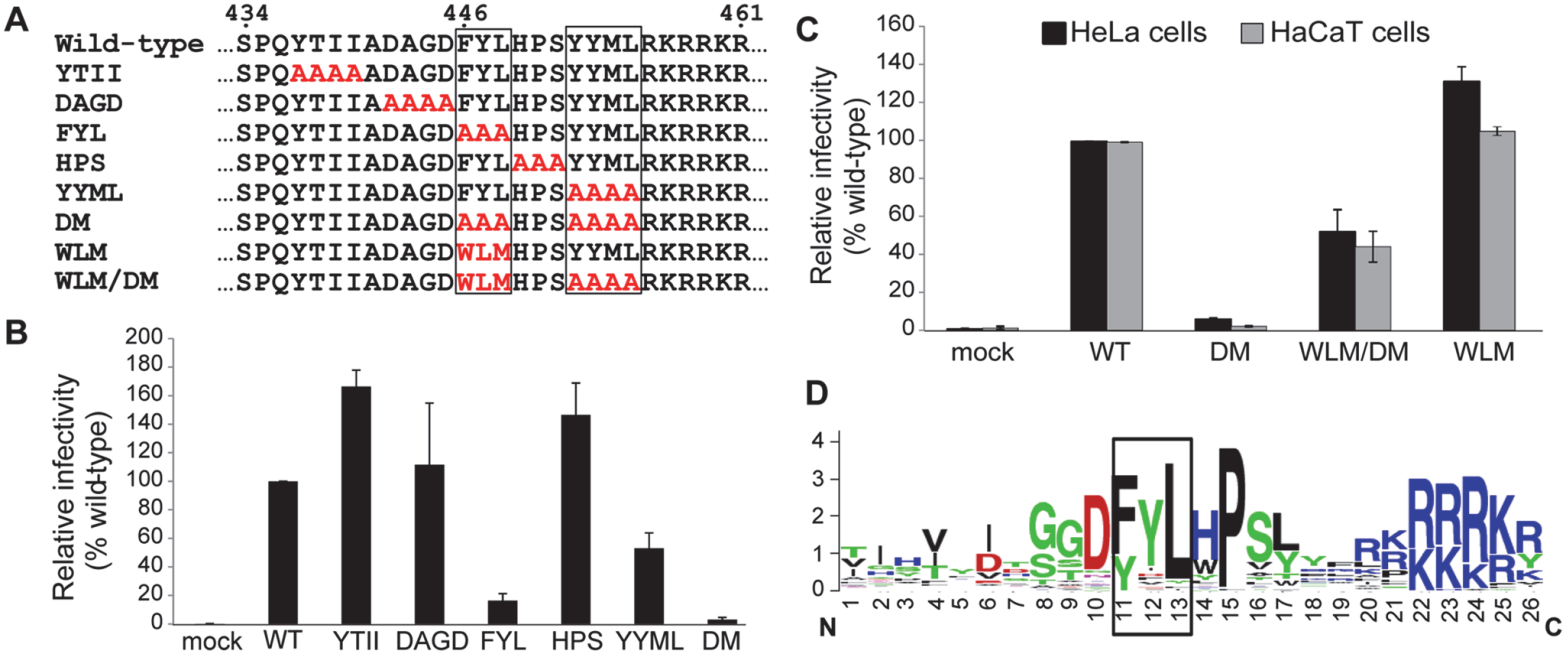
The carboxy-terminus of HPV16 L2 contains two potential retromer recognition motifs required for infectivity. **A**. Alanine scanning mutagenesis of the carboxy-terminus of the HPV16 L2 protein. The top line shows amino acids 434 to 461 of the wild-type L2 protein, with the potential retromer binding motifs in boxes. Amino acid substitutions in the mutants are shown in red. The double mutant (DM) has both potential retromer binding sites replaced with alanines; WLM contains a known retromer signal in place of FYL but retains the wild-type YYML sequence; WLM/DM contains the WLM substitution and the YYML to AAAA substitution. **B**. HA-tagged PsVs containing equal numbers of the GFP reporter plasmid (corresponding to MOI of approximately 0.5 for the wild-type) were added to HeLa cells, and 48h post-infection GFP-positive cells were counted by flow cytometry. WT indicates wild-type PsV. Multiple independent pseudovirus stocks were tested for each mutant. Results shown are averages of four independent experiments, with error bars showing standard deviation. **C**. HeLa (black bars) and HaCaT cells (grey bars) were infected with equal numbers of FLAG-tagged PsV (corresponding to MOI of 0.5 in each cell type for the wild-type virus). Forty-eight hours post-infection, GFP-positive cells were counted by flow cytometry. WLM/DM, the FYL/AAA mutation in the double mutant was replaced with Trp-Leu-Met; WLM, FYL to WLM mutation only. Results shown are averages of multiple independent experiments with multiple independent PsV stocks. **D**. L2 sequences of HPV types 1 to 45 were retrieved from the Papillomavirus episteme PaVE database (http://pave.niaid.nih.gov/) and trimmed to a 26 amino acid segment centered on the L in FYL (position 13). Sequence conservation is shown in a Sequence Logo plot (http://weblogo.berkeley.edu/logo.cgi). Conserved FYL and related sequences are shown in box.

Because retromer is required for the delivery of HPV16 to the Golgi apparatus and initiates endosome-to-Golgi transport of various cellular proteins, we hypothesized that a viral protein might bind to retromer. Inspection of the amino acid sequence of the HPV16 L2 protein revealed that its carboxy-terminal segment contains two short sequences, FYL and YYML, that resemble known retromer binding motifs, *e*.*g*., aromatic followed by any amino acid followed by leucine or methionine [ФXL/M], or phenylalanine or tryptophan followed by leucine followed by a valine or methionine [Trp/Phe-Leu-Met/Val] [37,38] (Fig. 1A). To determine if these sequences are important for HPV infection, we constructed alanine scanning mutations across this segment of L2 (Fig. 1A). Because it is difficult to introduce mutations into authentic HPV, we used pseudoviruses (PsVs) comprised of L1 and L2 encapsidating a reporter plasmid, which display the entry properties of authentic virus [39,40]. PsV containing wild-type L1, wild-type or mutant L2 with a C-terminal HA or FLAG tag, and a GFP or HcRed reporter plasmid were produced in 293TT cells [24,40]. The assembly of mutant capsids was confirmed by encapsidation of the reporter plasmid and electron microscopy, which revealed no morphologic differences from wild-type PsV (S1A Fig). In addition, when normalized by encapsidated reporter virus plasmids, wild-type and mutant pseudovirus preparations displayed a similar level of purity and contained similar levels of L1 and L2 (S1B and S1C Fig). HeLa cells were infected with wild-type and mutant PsV stocks containing the same number of encapsidated reporter plasmids, corresponding to a multiplicity of infection (MOI) of approximately 0.5 for wild-type, and successful infection was measured two days later by flow cytometry for GFP fluorescence. As shown in Fig. 1B, several mutants were competent to infect cells. Strikingly, however, the FYL/AAA mutant lacking one of the putative retromer binding sites showed a >80% reduction in infectivity, and the YYML/AAAA mutant lacking the other site showed an approximately 50% reduction. When the FYL and YYML mutations were combined to generate the double mutant (HPV16.L2DM), infectivity was essentially abolished. The double mutant showed a similar defect when the HA tag on L2 was replaced with a FLAG tag. The double mutant was also defective in HaCaT cells, a human skin keratinocyte cell line commonly used in HPV entry studies (*e*.*g*., [19,20,25]) (Fig. 1C), which also require retromer for efficient infection (S2 Fig).

The defect caused by mutations in the putative retromer binding sites in L2 suggests that retromer may interact with these sequences to promote HPV infection. To explore this possibility, we replaced FYL with the sequence tryptophan-leucine-methionine (WLM) (Fig. 1A), a retromer sorting signal from the cytoplasmic tail of the cation-independent mannose-6-phosphate receptor (CIMPR), a cellular cargo of retromer [37]. As shown in Fig. 1A and 1C, insertion of WLM into the double mutant to generate HPV16.L2WLM/DM partially restored the ability of HPV16.L2DM to infect HeLa and HaCaT cells, and a mutant containing WLM and the endogenous YYML sequence (HPV16.L2WLM) infected cells as well as wild-type PsV. Taken together, these results demonstrate that the putative retromer binding motifs in the carboxy-terminus of L2 are required for efficient infection and suggest that they act by binding to retromer. Alignment of the carboxy-terminal segment of the L2 protein from multiple HPV types showed that the FYL putative retromer binding site or a closely related sequence is highly conserved in all genera of HPV (Fig. 1D), but the YYML sequence is not. This correlates with the more dramatic defect caused by removal of the FYL sequence.

### Sequence from the L2 protein can act as a retromer sorting signal

**Fig 2:**
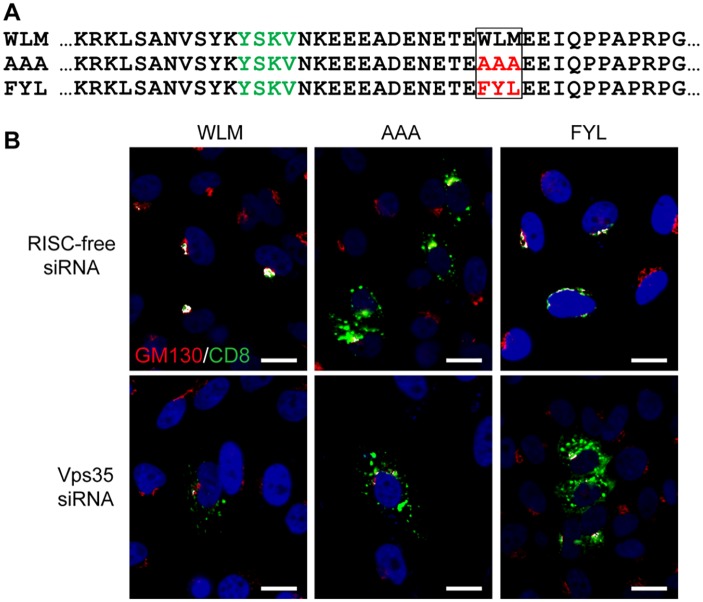
The L2 FYL sequence can act as a retromer sorting motif. **A**. Sequences of the membrane-proximal cytoplasmic tail of CD8-CIMPR fusion proteins used in this experiment. The YSKV endocytosis motif in CIMPR is in green, mutations in the WLM retromer sorting signal are in red. **B**. HeLa-M cells were transfected with a plasmid expressing the wild-type CD8-CIMPR fusion protein (WLM) or a mutant fusion protein with mutations replacing WLM (AAA or FYL) and with control RISC-free siRNA or siRNA targeting retromer subunit Vps35, as indicated. After 24 hours, live cells were incubated with anti-CD8 for three hours, and localization of the construct was assessed by co-immunofluorescence with Golgi marker GM130. CD8-CIMPR fusion protein, green; GM130, red; nuclei, blue. A single plane in the Z-dimension is shown in each panel. Overlap between fusion protein and Golgi marker GM130 is pseudocolored white. The size bars on these and all subsequent images are 20 μM. (We note that HeLa-M cells have larger nuclei than the HeLa cells used in other experiments.) The component images used to generate this figure are shown in S4 Fig. Similar results were obtained in three independent experiments.

To test directly if the sequence FYL can act as a retromer sorting signal, we used an antibody internalization assay to determine if FYL is able to replace the endogenous sorting signal in a cellular retromer cargo [41]. The extracellular and transmembrane domains of the cell-surface protein CD8 were fused to the 160-amino acid cytoplasmic tail of CIMPR, which contains an endocytosis motif and the WLM retromer sorting signal identified by Seaman and colleagues [37] (Fig. 2A). This signal mediates retromer-dependent trafficking of CIMPR from the endosome to the Golgi apparatus. HeLa cells were transfected with a plasmid expressing CD8-CIMPR containing a wild-type or mutant WLM sequence, and after 24 hours, live non-permeabilized cells were incubated with a CD8 antibody for three hours at 37°C. During this incubation, the CD8-CIMPR fusion protein containing the endogenous WLM sorting signal was endocytosed and trafficked to the TGN, which was scored by co-localization with the Golgi marker GM130. As previously reported [37], substitution of the WLM sequence with AAA abolished Golgi trafficking, resulting in a punctate distribution of the fusion protein throughout the cytoplasm. Strikingly, replacement of WLM with FYL restored Golgi localization (Fig. 2B). Furthermore, siRNA-mediated knockdown of the Vps35 retromer subunit (S3 Fig) eliminated Golgi localization of the WLM and FYL CD8-CIMPR fusion proteins in the antibody internalization assay and resulted in dispersed punctate distribution of the fusion proteins (Fig. 2B). These results demonstrate that FYL can act as a retromer sorting signal in a standard trafficking assay.

### Retromer is required for exit of HPV from the early endosome

The experiments described above imply that retromer sorting signals in the L2 protein are involved in HPV trafficking during virus entry. To determine the role of retromer and the putative retromer sorting motifs in HPV infection, we conducted experiments in infected cells. To first rule out the possibility that the defect in infectivity caused by the L2 mutations was due to an inability of the mutant to enter cells or undergo disassembly, we infected HeLa cells with PsV containing L2 with a carboxy-terminal FLAG epitope tag (designated HPV16.L2F). The FLAG tag is constitutively exposed on the surface of capsids and does not inhibit infectivity nor affect entry requirements, as assessed by sensitivity to several genetic and pharmacologic inhibitors of entry [24]. We stained cells at early times after infection with the anti-FLAG antibody or with the 33L1–7 antibody, which recognizes an epitope on the L1 protein that is inaccessible in intact capsids and reacts with L1 only after the capsid has entered cells and disassembly has begun [20,29,42]. Neither antibody stained uninfected cells, but cells infected with PsV containing wild-type or double mutant L2 displayed similar punctate intracellular staining with both antibodies (S5 Fig), demonstrating that the inability of the L2 double mutant to infect cells is not due to a defect in internalization or initiation of capsid disassembly.

**Fig 3:**
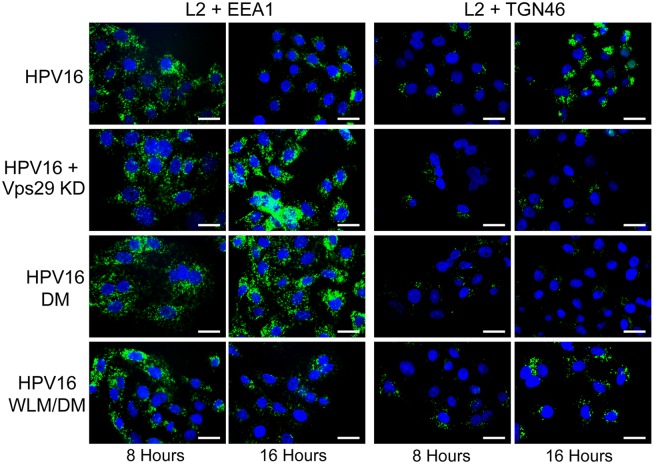
Retromer activity and potential retromer binding sites in L2 are required for exit from the early endosome and entry into the *trans*-Golgi network. HeLa cells were transfected with siRNA targeting retromer subunit Vps29 (Vps29 KD) or left untreated. 48 hours later, cells were infected with FLAG-tagged wild-type HPV16, HPV16.L2DM or HPV16.L2WLM/DM PsV at MOI of 50 (according to reporter plasmid normalization). Eight or 16 hours post-infection, cells were fixed, permeabilized, and incubated with an anti-FLAG antibody and an antibody recognizing EEA1 (early endosome marker) or TGN46 (TGN marker). PLA was performed to assess L2 in proximity to the cellular marker (green). Nuclei are stained blue with DAPI. A single plane in the Z-dimension is shown in each panel. Similar results were obtained in multiple independent experiments with independent PsV stocks.

**Fig 4:**
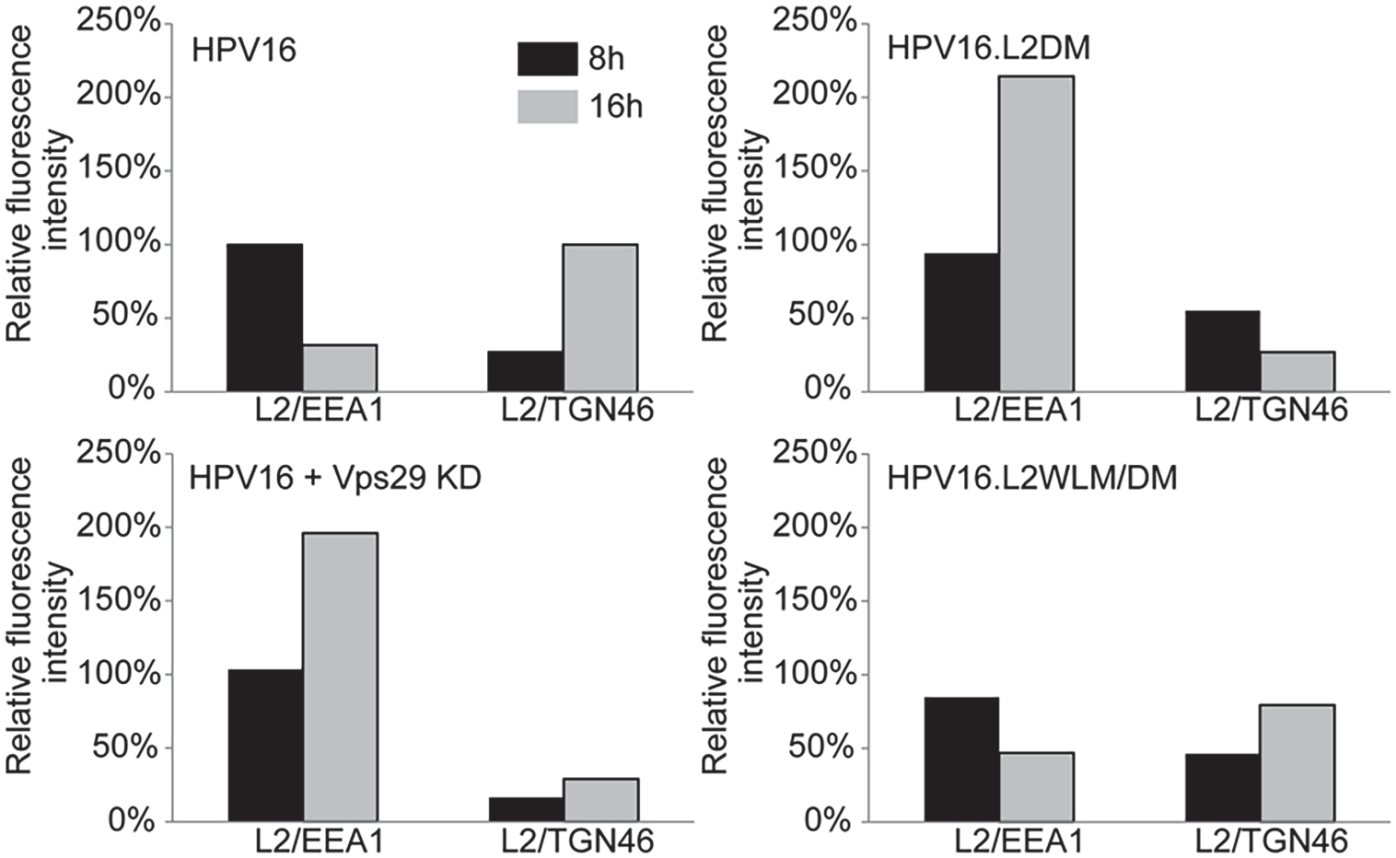
Quantitation of HPV trafficking in the absence of retromer activity or binding. Images obtained as in Fig. 3 were processed by Blobfinder software to determine the PLA fluorescence intensity per cell in each sample. The average intensity for EEA1/L2 samples was normalized to the control sample infected with wild-type PsV at eight hours post-infection, and the average intensity for TGN46/L2 samples was normalized to the control sample infected with wild-type PsV at 16 hours post-infection. Black bars, eight hours; grey bars, 16 hours. The results show quantitation of a representative experiment. Because of the inherent variability of PLA assays, it was not possible to directly compare independent experiments, but similar results were obtained in two additional independent experiments.

To determine if retromer knock-down impairs exit of HPV PsV from the endosome, we used the proximity ligation assay (PLA), a specific immune-based detection system in which a fluorescent signal is generated only when two proteins of interest are nominally within 40nm [43,44]. PLA was used to determine if L2 is in close proximity to the early endosome marker EEA1 or the *trans*-Golgi marker TGN46 during entry. At eight and 16 hours post-infection with HPV16.L2F, we incubated fixed and permeabilized cells with anti-FLAG and the antibody recognizing the cellular component and then processed the samples for PLA. As expected, PLA did not generate signals in uninfected cells. When HeLa or HaCaT cells were infected with wild-type HPV16.L2F and stained for L2-FLAG in proximity to EEA1, a PLA signal was detected at eight hours post-infection, and by 16 hours the PLA signal associated with this compartment was reduced (Figs. 3, 4, and S6). In contrast, there was little L2/TGN46 PLA signal in the Golgi at eight hours post-infection, but there was abundant signal by 16 hours. These results indicate that virus enters the early endosome by eight hours after infection, but by 16 hours it has transited through this compartment and arrived at the Golgi. HPV trafficking was strikingly different in retromer knock-down cells. In this experiment, HeLa and HaCaT cells were transfected with siRNA targeting the retromer subunit Vps29, infected with HPV16.L2F two days later, and subjected to PLA at various times post-infection. An L2/EEA1 PLA signal was observed in both control and retromer knock-down cells eight hours after infection, confirming that retromer is not required for virus endocytosis. However, at 16 hours post-infection, the L2/TGN46 PLA signal was essentially undetectable, whereas there was a striking increase in the L2/EEA1 PLA signal (Figs. 3, 4, and S6). This is in marked contrast to infected parental cells, which contained little endosomal L2 at 16 hours after infection. These experiments demonstrate that retromer knock-down in both HeLa and HaCaT cells causes the accumulation of L2 in the early endosome and prevents the arrival of L2 into the Golgi.

### Retromer binding motifs in L2 are required for export from the early endosome

If the non-infectious phenotype of HPV16.L2DM is due to interference with retromer recognition, this mutant should fail to exit from the early endosome in cells with intact retromer function. To test this, we infected HeLa and HaCaT cells with FLAG-tagged HPV16.L2DM and used PLA to assess localization of incoming virus. As shown in Figs. 3, 4, and S6, an L2/EEA1 PLA signal was observed in cells infected with HPV16.L2DM at eight hours after infection. At 16 hours post-infection, an L2/TGN46 PLA signal was not observed, while the L2/EEA1 PLA signal markedly increased. Thus, mutation of the putative retromer binding motifs in the carboxy-terminal segment of L2 is functionally equivalent to knocking down retromer function. Importantly, when WLM was inserted into HPV16.L2DM at the original position of FYL (to generate HPV16.L2WLM/DM), a substantial restoration of the L2/TGN46 PLA signal was observed at 16 hours after infection in both cell types, together with a reduction in the EEA1 signal at this time, confirming that the WLM retromer motif restores exit of L2 from the early endosome and trafficking to the Golgi. Similar results were obtained with multiple independent stocks of HPV16.L2DM and HPV16.L2WLM/DM. These results strongly suggest that the trafficking defect displayed by the double L2 mutant is due to impaired retromer binding.

### Mutations in the retromer motifs inhibit association between retromer and L2 in infected cells

**Fig 5:**
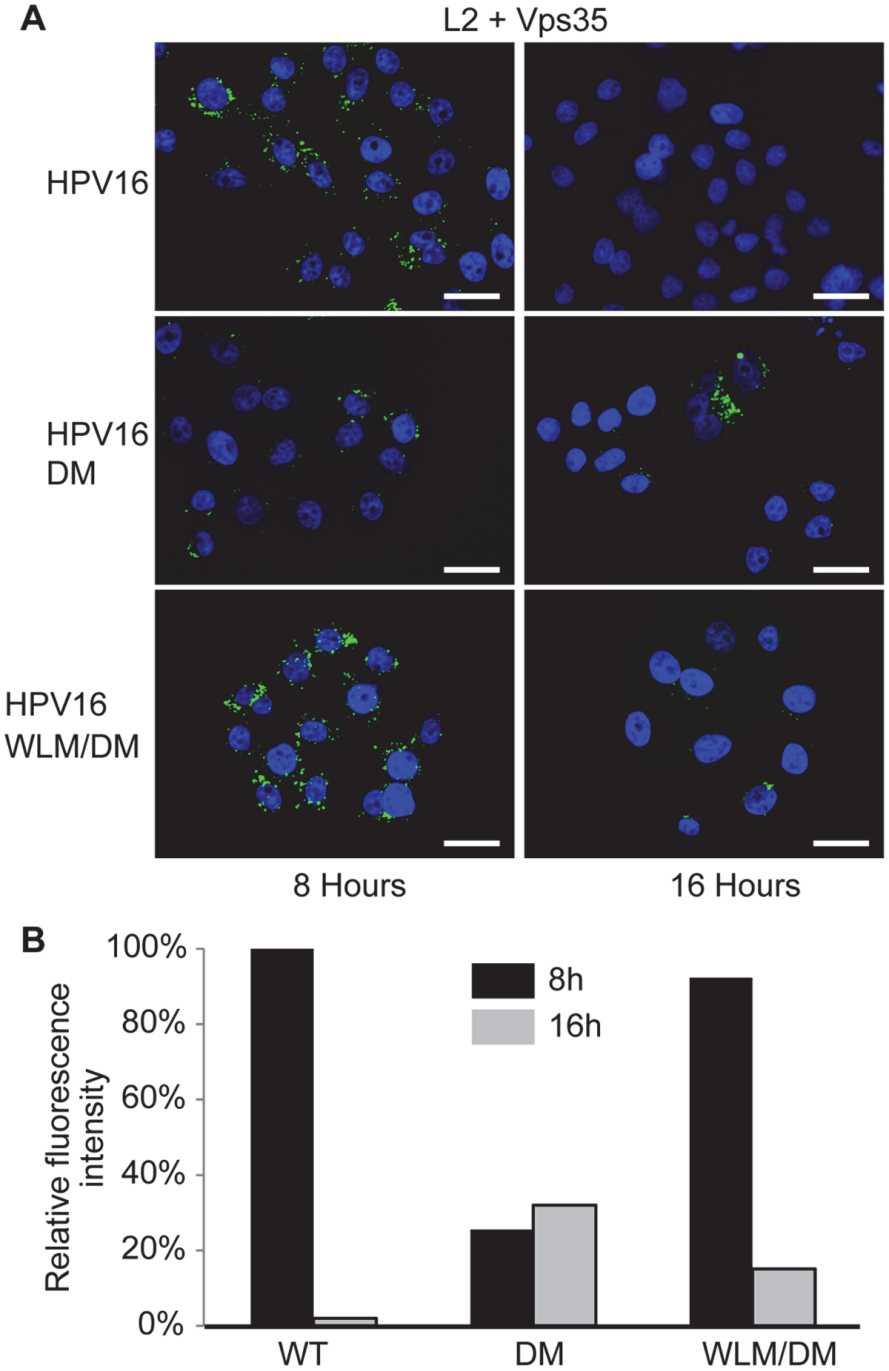
Retromer sorting signals in L2 C-terminus are important for its association with retromer during entry. **A**. HeLa cells were infected with FLAG-tagged HPV16, HPV16.L2DM or HPV16.L2WLM/DM PsV at MOI of 50. Eight or 16 hours post-infection, cells were fixed, permeabilized, and incubated with an anti-FLAG antibody and an antibody recognizing retromer subunit Vps35. PLA was performed to assess proximity of L2 and Vps35 (green). Nuclei are stained blue with DAPI. A single plane in the Z-dimension is shown in each panel. **B**. Images obtained as in panel A were processed by Blobfinder software to determine the PLA fluorescence intensity per cell in each sample. The average intensity was normalized to the control sample infected with wild-type PsV at eight hours post-infection. Black bars, eight hours; grey bars, 16 hours. The results show quantitation of a representative experiment. Similar results were obtained in two additional independent experiments.

Recognition of cargo is a major factor underlying retromer recruitment to membranes [45]. To test whether the trafficking defect displayed by the L2 double mutant correlated with impaired association between L2 and endogenous retromer in infected cells, we performed PLA with anti-FLAG and anti-Vps35. HeLa cells were infected with FLAG-tagged HPV16 PsV containing wild-type L2, L2DM, or L2WLM/DM, and PLA was performed at eight and 16 hours post-infection. As shown in Fig. 5, a PLA signal was observed for wild-type L2 and retromer at eight hours. This signal decreased substantially by 16 hours, consistent with a transient association between L2 and retromer as HPV exits the early endosome. In contrast, at eight hours after infection with HPV16.L2DM, the PLA signal for L2 and Vps35 was decreased by 70% compared to cells infected with the wild-type PsV. The signal persisted at this level at 16 hours, despite the markedly increased amount of the mutant L2 protein in the early endosome at this time. Replacing FYL in the double mutant with WLM restored transient association of the L2 protein with retromer. Taken together, these results indicate that mutations that impair the association of L2 with retromer in infected cells also impair L2 export from the early endosome and demonstrate that the WLM sequence restores association between L2 and retromer, as well as restoring endosome exit.

### The carboxy-terminal segment of the L2 protein binds directly to retromer

**Fig 6:**
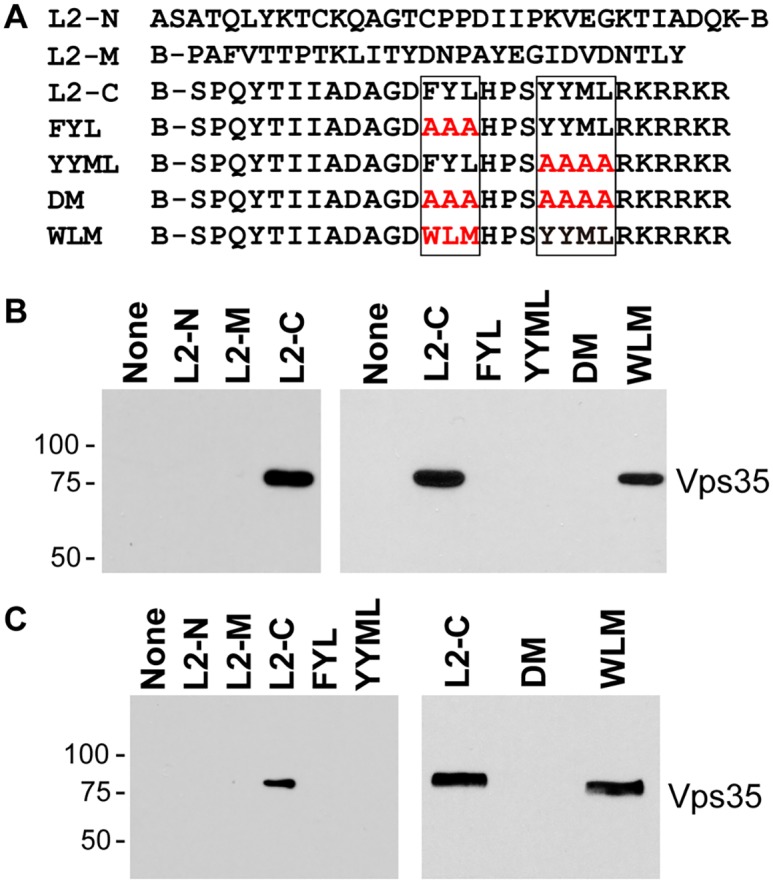
Binding of carboxy-terminal L2 peptides to retromer. **A**. The top lines show the sequences of biotinylated peptides, where B indicates position of biotin. Putative retromer recognition motifs in the carboxy-terminal peptide are shown in boxes. Mutant versions of the carboxy-terminal peptide are also shown, with the mutations in red. **B**. Left panel. L2-N, L2-M, or L2-C peptide was incubated with uninfected HeLa cell RIPA lysate. The samples were analyzed by streptavidin pull-down, SDS-PAGE, and immunoblotting with an anti-Vps35 antibody. Molecular weight markers in kDa are shown at the left. Right panel. The wild-type or a mutant carboxy-terminal peptide were incubated with uninfected HeLa cell HEPES lysate and processed as in panel A. Similar results were obtained in three or more independent experiments. **C**. Experiments performed as in panel B with HaCaT cell RIPA lysates.

To determine if the carboxy-terminal segment of L2 is sufficient to bind to retromer, we conducted *in vitro* pull-down experiments. First, we employed biotinylated peptides, one from the amino-terminal portion of the L2 protein, one from the middle of the L2 protein, and one from the carboxy-terminal portion, including the two putative retromer binding sites (Fig. 6A). These peptides were incubated with detergent lysates of uninfected HeLa and HaCaT cells, and cellular proteins that bound to the peptides were collected on streptavidin beads, subjected to SDS-polyacrylamide gel electrophoresis and immunoblotted for retromer subunits. The carboxy-terminal peptide containing the retromer motifs precipitated endogenous Vps29 and Vps35, whereas the other two peptides were devoid of retromer binding activity (Figs. 6B and 6C, left panels, and S7).

We also conducted pull-down experiments with carboxy-terminal peptides containing mutations in the retromer binding motifs. As shown in Fig. 6B and 6C, mutations in either retromer motif eliminated retromer binding in HeLa and HaCaT cell lysates, as did mutation of both sites. In some experiments, slight binding to the YYML/AAAA mutant was observed (S8 Fig), but binding to the FYL/AAA mutant was never detected, suggesting that the FYL mutation causes a more severe defect in retromer binding, consistent with the more dramatic defect in infection caused by the FYL mutation. Importantly, replacement of FYL with the WLM retromer motif restored a significant level of retromer binding in extracts of either cell type (Fig. 6). Taken together, these results show that the retromer sorting motifs in the C-terminus of L2 bind to endogenous retromer *in vitro*, and that mutations that inhibit infectivity and endosome exit interfere with retromer binding.

**Fig 7:**
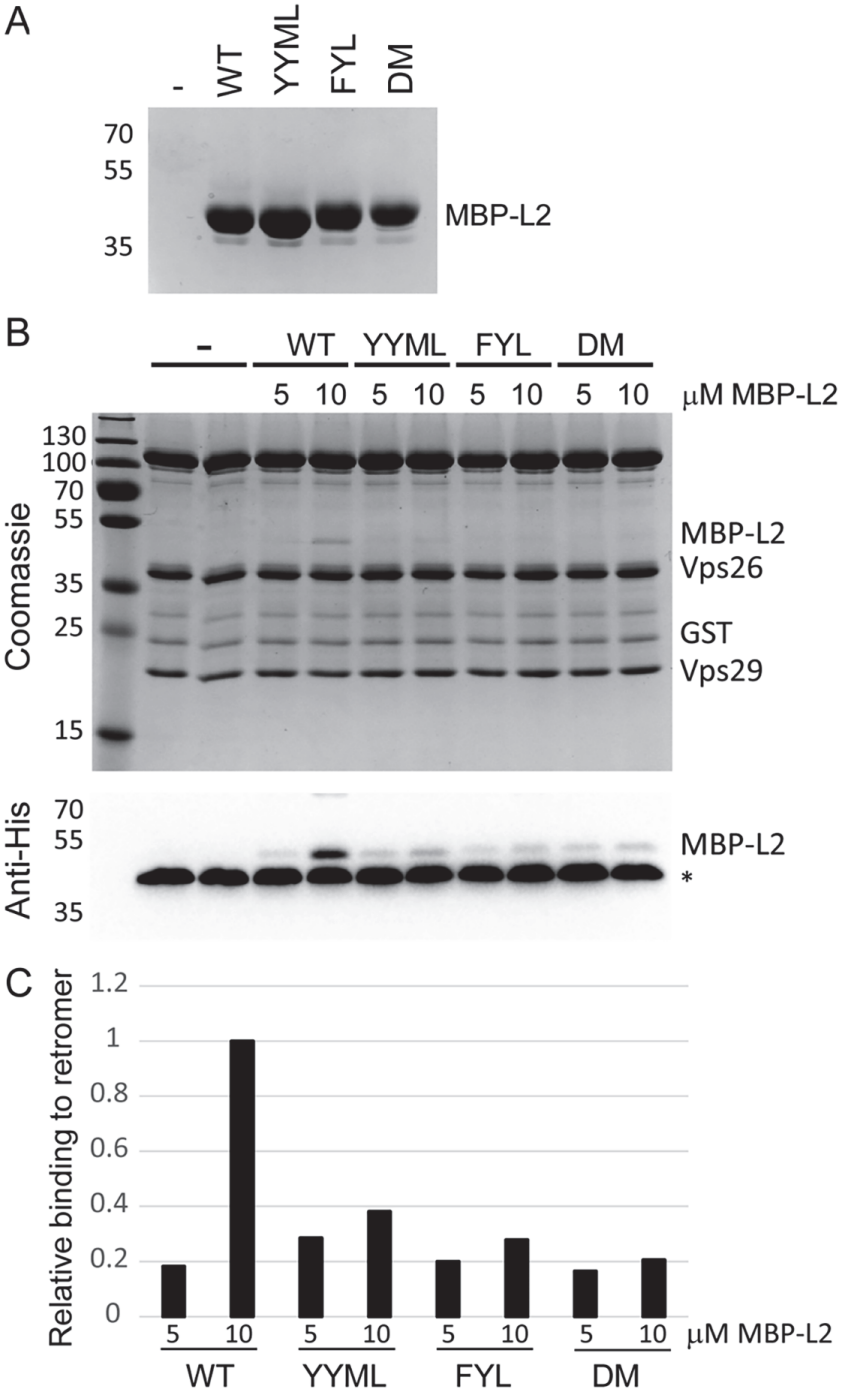
L2 retromer motifs mediate direct binding to retromer. **A**. MBP-L2 fusion proteins containing the wild-type or mutant carboxy-terminal segment of L2 were purified from *E*. *coli* and subjected to SDS-PAGE and staining with coomassie brilliant blue. Molecular weight markers are shown on the left. **B**. 5 or 10μM purified MBP-L2 fusion proteins were bound to purified retromer trimer, eluted, and subjected to SDS-polyacrylamine gel electrophoresis. Proteins were visualized by coomassie brilliant blue staining (top panel) or immunoblotting for the His-epitope on the MBP fusion protein (bottom panel). The band migrating at ~40 kDa indicated by the asterisks in all lanes in the immunoblot is Vps26, which cross-reacts with the anti-His antibody. Similar results were obtained in two independent experiments. **C**. The immunoblotting results in panel B were quantified and are plotted relative to binding of retromer to 10μM wild-type MBP-L2 fusion protein.

To determine if retromer directly recognizes L2, we tested whether the carboxy-terminus of L2 was able to bind to active human retromer assembled from individual Vps26, Vps29, and Vps35 subunits purified from *E*. *coli* and immobilized on glutathione resin. We previously showed that retromer assembled in this way bound to the cellular retromer cargo, DMT1-II [45]. A 24-amino acid wild-type or mutant segment of L2 containing the retromer binding sites was fused to poly-histidine-tagged maltose binding protein (MBP), which was also expressed and purified from *E*. *coli* (Fig. 7A). The L2 fusion protein was incubated with immobilized retromer, and the L2 fusion protein bound to retromer was eluted and detected following SDS-PAGE. As shown in Figs. 7B and 7C, retromer captured the L2 fusion protein containing the carboxy-terminal segment of the wild-type L2 protein, indicating that retromer and this segment of L2 interact directly. In contrast, the FYL and YYML alanine substitutions, alone or in combination, drastically decreased retromer binding. Thus, the carboxy-terminal segment of the L2 protein binds directly to retromer via sites required for exit from the early endosome.

## Discussion

Viruses utilize cellular machinery to reach the site of viral genome replication. Therefore, studies of virus entry not only reveal important features of the virus life cycle, but also elucidate the mechanisms cells use to ensure that cellular components are present in their proper intracellular locations. We previously identified retromer as a factor required for trafficking of HPV16 to the Golgi apparatus during infection [22], but our published experiments did not determine if retromer plays a direct or indirect role in HPV infection. HPV is a non-enveloped virus that lacks transmembrane proteins and is present in the endosomal lumen early during entry. Thus retromer, which is present in the cytoplasm and transports transmembrane proteins, could act indirectly on a cellular cargo to support HPV entry, or it could recognize the HPV capsid in an unconventional manner. The experiments reported here reveal that the papillomavirus capsid is a new class of retromer cargo and that a direct interaction between retromer and the L2 minor capsid protein is required for L2 to exit the endosome and traffic to the Golgi. Because L2 is closely associated with the viral genome throughout the entry process and manipulations that interfere with L2 trafficking also inhibit infectivity, we conclude that the observed behavior of L2 reflects the behavior of the viral components required for infectivity.

Several lines of evidence demonstrate that L2 is a retromer cargo. Retromer knock-down causes HPV L2 to accumulate in the early endosome in HeLa and HaCaT cells. Furthermore, the carboxy-terminal segment of the L2 protein contains short sequences that resemble known retromer binding motifs, and mutations in these motifs interfere with the ability of L2 to associate with retromer in infected cells and inhibit the export of PsV from the early endosome and its delivery to the Golgi apparatus in HeLa and HaCaT cells. Importantly, these defects are rescued by replacement of the major retromer binding site in L2 with a retromer sorting signal from a cellular protein. In addition, this L2 sequence can function in a standard retromer sorting assay. Finally, *in vitro* binding studies showed that these sites in the L2 protein bind directly to retromer. Taken together, these results demonstrate that a direct interaction between the carboxy terminus of the L2 protein and retromer is required for exit of L2 from the early endosome and subsequent entry into the Golgi during HPV16 entry. These findings strongly suggest that retromer sorts HPV into an endosome-derived transport vesicle that ferries HPV or a subviral structure containing L2 and viral DNA to the TGN. Elsewhere, we showed that inhibition of γ-secretase blocks HPV trafficking after export from the early endosome but before delivery to the Golgi and the ER [24], indicating that γ-secretase is required in HPV entry after retromer action.

The peptide and fusion protein pull-down experiments suggest that both the FYL and YYML motifs are required for efficient retromer binding *in vitro*, although YYML appears less important (S8 Fig). However, the more dramatic infectivity defect caused by the FYL mutation and the lack of YYML conservation indicates that the FYL sequence is more important in cells and during natural infection.

FYL or a closely related sequence is present in the same position in all sequenced HPV L2 proteins examined and in the great majority of animal papillomaviruses, implying that the ability of retromer to recognize the L2 protein and export it from the early endosome arose early during papillomavirus evolution and remains an important feature of the virus life cycle. Some positions flanking FYL are also highly conserved, but they are not required for HPV16 entry in the assays used here. We also note that FYL and YYML do not match the canonical CIMPR retromer binding motif, Trp/Phe-Leu-Met/Val. FYL does appear similar to the ФXL/M motif in the mammalian iron transporter, DMT1-II [38]. However, comparison of this sequence in numerous HPV types reveals important differences. Although the ФXL/M motif can accommodate tryptophan in the first position, in 45 HPV L2 proteins examined, ~70% contain phenylalanine at this position, ~30% contain tyrosine, and none contain tryptophan. At the second position, ~75% HPV contain tyrosine and only one contains leucine, while wild-type DMT1-II contains leucine. Thus, although the HPV L2 and DMT1-II motifs are related, the examination of numerous HPV types suggests that evolution has selected specific residues at these positions in the viral protein that differ from the sequence in DMT1-II. Similar non-canonical retromer binding motifs may exist in additional retromer cargos from cells or other viruses.

The L2 protein may be analogous to capsid proteins of other non-enveloped viruses that undergo conformation changes that expose hydrophobic peptides that insert into cell membranes and disrupt them to allow capsid entry into the cytoplasm [46,47]. We propose that the carboxy-terminal segment of L2 causes a more subtle perturbation of membrane structure, allowing it to protrude through the endosomal membrane where it is recognized by retromer on the cytoplasmic face of the membrane. Cells may contain other similar, as-yet-unrecognized, non-conventional retromer cargos, and other viruses may use a similar entry mechanism.

A peptide derived from the carboxy-terminal segment of L2 including YYML and an adjacent, conserved highly basic sequence exhibits membrane bilayer-disrupting activity *in vitro* and can mediate integration of a reporter protein into cell membranes [31]. This peptide disrupts membranes only at low pH [31]. Therefore, endosome acidification may trigger its penetration through the endosomal membrane during virus entry. Such behavior may explain at least in part the requirement for endosome acidification during HPV entry [17,18,23].

It is possible that the carboxy-terminal segment of L2 cooperates with another segment of the L2 protein exposed on the surface of the capsid to mediate membrane penetration and/or retromer recognition in intact cells. The L2 amino-terminus contains a transmembrane-like domain required for infectivity [34]. This L2 segment may stabilize the association of L2 with the endosomal membrane or retromer or facilitate the passage of the carboxy-terminal segment containing the retromer binding sites through the membrane. In addition, a central segment of L2 binds SNX17, another cytoplasmic protein required for efficient infection [30], implying that this L2 segment is also accessible to the cytoplasm in some situations. Interestingly, SNX17 is reported to cooperate with retromer in mediating recycling of the Notch ligand, Jag1a [48]. Finally, the basic sequence adjacent to YYML resembles motifs present in cell-penetrating peptides, which can carry protein cargo across membranes into cells [49]. Further analysis will determine if any of these sequences play a role in retromer action during HPV infection.

In summary, these studies elucidate the role of retromer in HPV entry, demonstrating that it binds directly to the HPV capsid and mediates export of this unconventional cargo from the early endosome on its journey into the cell. We propose that retromer recognition in this system involves a novel mechanism whereby a segment of a minor capsid protein of a non-enveloped virus protrudes through a cellular membrane into the cytoplasm. Further analysis of the role of retromer in HPV16 infection will provide new insights into virus entry, retromer function, and intracellular trafficking.

## Materials and Methods

### Cells

HaCaT cells are a spontaneously immortalized line of human skin keratinocytes obtained from G. Paolo Dotto (Massachusetts General Hospital) [50]. 293TT cells were obtained from Dr. Christopher Buck (NIH). HeLa-Sen2 cells (designated here HeLa cells) are a previously described cloned strain of HeLa cells that infects efficiently with SV40 and HPV16 pseudovirus [51]. HeLa-M cells, a strain of HeLa-S3 cells that transfects efficiently, were obtained from Walther Mothes (Yale University). All cells were cultured in Dulbecco’s MEM (DMEM) with 10% fetal bovine serum (FBS), 10mM L-glutamine, 10mM HEPES pH 7.2 and standard antibiotics (Pen/Strep).

### Viruses and plasmids

HPV16-GFP pseudovirus containing an HA tag at the C-terminus of L2 (designated here HPV16.L2HA) was generated by using a plasmid obtained from Patricia Day. We also used PsV designated HPV16.L2F, which contains an L2 protein with a carboxy-terminal 3xFLAG-tag constitutively exposed on the surface of the capsid [24]. The HPV16 L2 C-terminal mutants were produced in either the HA- or the FLAG-tagged p16sheLL expression plasmid using the QuickChange site-directed mutagenesis system and the primers listed in S1 Table. The L1 and L2 genes in each mutant were sequenced in their entirety. Mutations in the retromer sorting motif in the CD8-CIMPR fusion protein were inserted into a plasmid expressing the wild-type fusion protein (a gift from Matthew Seaman, Cambridge Institute for Medical Research). siRNA targeting Vps26, Vps29, and Vps35 and the control scrambled siRNA were purchased from Dharmacon (Lafayette, CO). Sequences of all oligonucleotides used in this study are listed in S1 Table.

pCINeo-GFP plasmid (obtained from Christopher Buck (NIH) and pCAG-HcRed plasmid (purchased from Addgene, Plasmid 11152) were used as reporter plasmids. Pseudoviruses were produced by co-transfecting 293TT cells with a p16sheLL plasmid expressing L1 and wild-type or mutant L2 and a reporter plasmid, and purified by density gradient centrifugation in OptiPrep (Sigma-Aldrich, #D1556) as previously described [22,40]. Encapsidated GFP or far-red genomes were quantified by qPCR as described [52,53]. Briefly, 5 μl of each pseudovirus preparation was treated with 4 μl of RQ1 DNAase (Promega, M6101) in 100 μl DNAase buffer (50mM Tris HCl pH 7.6, 10mM MgCl_2_) for one hour at 37°C. The DNAase was inactivated by incubation at 75°C for 30 min, followed by the addition of 50 μg of proteinase K (PK, Roche) for one hour at 37°C, in PK buffer (10mM Tris HCl pH 8.0, 10mM EDTA, 0.25% SDS) [52]. DNA was isolated using a PCR purification kit, and the number of encapsidated genomes was determined by qPCR using primers for the GFP or far-red gene, using a 10-fold serial dilution of pCINeo-GFP or pCAG-HcRed plasmid (10^9^ to 10^3^ genomes/μl) analyzed on the same plate as a standard. Encapsidated genomes for all of the PsV stocks used in any one experiment were quantified in parallel. To examine the purity and content of L1 and L2 in PsVs, Optiprep-purified PsV preparations containing FLAG-tagged L2 were denatured in SDS-Laemmli sample buffer (10^8^ packaged reporter plasmid genomes/lane) and electrophoresed on a SDS-10% polyacrylamide gel for 1.5h at 150V. Proteins were subjected to silver staining or transferred from the gel to PVDF membrane (Millipore Immobilon, 0.2 μm, ISEQ15150), which was probed with 0.5 μg/mL primary anti-L1 (BD Pharmingen, 554171) or anti-FLAG (Sigma, F3165) antibody. Following incubation with 1:10,000 dilution of horseradish peroxidase-coupled secondary antibodies, bands were visualized by luminescence (SuperSignal West Pico, Thermo Scientific, 34080).

### Electron microscopy of purified pseudovirus

Freshly glow-discharged 200 mesh Formvar/carbon-coated copper grids (Electron Microscopy Services, CG200-Cu) were inverted on drops of gradient-purified PsV diluted 1:9 in phosphate-buffered saline (PBS), and virus allowed to adsorb for five minutes. The grids were washed twice in deionized water and stained by two one-minute incubations on drops of Nano-W (Nanoprobes, Nephank, NY) before removing excess stain by gentle blotting with Whatman #1 filter paper. The grids were air-dried before viewing on a FEI Tecnai Biotwin transmission electron microscope at 80Kv. Images were taken using a Morada CCD camera and iTEM (Olympus) software, and ImageJ was used to provide sizing information based on a scale bar embedded in the images.

### Infectivity assays

5×10^4^ HeLa or HaCaT cells were plated in 12-well plates. Cells were infected with wild-type HPV16 PsV at MOI ~0.5 GFP-transducing units per cell (*i*.*e*., enough virus to result in GFP expression in one-half of the cells as assessed by flow cytometry on a BD Biosciences FACSCalibur flow cytometer 48 hours post-infection). The number of packaged wild-type reporter plasmids required to achieve this MOI in unmanipulated cells was quantified by qPCR, and an equivalent number of mutant genomes were used to infect cells. Depending on the experiment, 150–300 reporter plasmid genomes per cell resulted in an MOI of ~0.5 for wild-type pseudovirus in HeLa cells; approximately five-fold more virus was required to attain this MOI in HaCaT cells. In some experiments, cells were transfected with siRNA prior to infection. To confirm retromer knock-down, 5×10^5^ cells were plated in a 6-well plate and reverse-transfected with 40nM siRNA targeting Vps26, Vps29, or Vps35. Forty-eight hours later, cells were lysed in sample buffer, electrophoresed, and analyzed by immunoblotting for Vps35.

### Immunofluorescence experiments

3 to 5×10^4^ HeLa cells were plated in eight-well chambered glass slides and infected the next day with wild-type PsV at MOI 20 or mutant PsV containing the same number of reporter plasmid genomes. (The lower sensitivity of immunofluorescence or PLA [see below] compared to reporter gene expression necessitated an MOI of 20 or 50 to visualize virus components.) Eight hours post-infection, the cells were fixed for 15 min at room temperature with 4% paraformaldehyde (Electron Microscopy Sciences, #15710), washed with PBS and then permeabilized with 0.5% Triton X-100 for 20 min at room temperature in PBS. The cells were blocked for one hour in 1% bovine serum albumin and 3% goat serum, and immunostained with 1:200 33L1–7 (obtained from Martin Sapp (LSU)). After extensive washes, AlexaFluor 594-conjugated goat anti-mouse secondary antibodies were added at 1:300 dilution for 40 minutes at room temperature. Nuclei were stained with 1:100 dilution of 4′,6-diamidino-2-phenylindole (DAPI), cells were washed extensively, and slides were mounted in Prolong gold anti-fade (Molecular Probes). Images were recorded on a ZEISS Axiovert 200 inverted fluorescent microscope using appropriate filters processed with ImageJ.

### CD8-CIMPR antibody capture experiment

3×10^4^ HeLa-M cells in 8-chambered glass slides were transfected with 10nM Vps35 or RISC-free siRNA. Twenty-four hours later, the Trans-IT HeLaMONSTER reagent (Mirus Bio) was used to transfect cells with 1 μg of a plasmid expressing a CD8-CIMPR fusion protein containing WLM (wild-type), AAA, or FYL. Twenty-four hours later, live non-permeabilized cells were incubated at 37°C with a 1:400 dilution of an antibody that recognizes the extracellular domain of CD8 (Ancell, 153–020). After three hours, the cells were fixed for 15 min at room temperature with 4% Formalde-Fresh, permeabilized with 0.5% Triton X-100 for 20 min, and then blocked with 5% donkey and goat serum for 30 minutes at room temperature. The cells were then stained with anti-GM130 (Abcam, ab52649 [1:200]) overnight at 4°C, washed five times with the blocking solution, and incubated with conjugated secondary antibody (Life Technologies [1:500]) for 30 minutes at room temperature. Cells were mounted with Duolink *in situ* Mounting Medium with DAPI, imaged on a ZEISS Axiovert 200 inverted fluorescent microscope and processed with Image J.

### Proximity ligation assay

5×10^4^ HeLa cells grown overnight on glass coverslips in 24-well plate were transfected with siRNA targeting retromer subunit Vps29 or control scrambled siRNA with Lipofectamine RNAi Max reagent (Life Technologies, Carlsbad, CA) 48 hours prior to infection. The cells were then infected with wild-type or mutant HPV16.L2F at MOI of 50 (according to genome normalization), fixed with 4% Formalde-Fresh at eight or sixteen hours post-infection, and permeabilized with 1% saponin for one hour at room temperature. The cells were incubated with anti-FLAG mouse antibody (Sigma, F3165 [1:1000]) to label L2 and an antibody recognizing EEA1 (Cell Signaling, C45B10 [1:100]) or TGN46 (Abcam, ab50595 [1:200]). Alternatively, cells were incubated with anti-FLAG rabbit antibody (Cell Signaling, 2368 [1:500]) and anti-Vps35 antibody (Abcam, 57632 [1:500]). PLA was performed with Duolink reagents from Olink Biosciences (Uppsala, Sweden) as described [22,54]. Briefly, samples were incubated with a pair of suitable PLA probes at 1:5 in a humidified chamber for one hour and processed for ligation for 30 min at 37°C. DNA was then amplified with fluorescent substrates for 100 min at 37°C. The nuclei were stained by incubation with 5μg/ml DAPI for 10 min and images were acquired as described above. Approximately 100 nuclei were imaged in each sample. The images were processed with ImageJ and quantitatively analyzed with BlobFinder software to measure total fluorescence intensity in each sample. The average fluorescence intensity per cell in each sample was normalized to the control sample as indicated in each experiment. All the experiments were done independently three times with similar results, and one representative experiment is shown.

### Peptide pull-down experiments

Peptides shown in Fig. 6 were purchased from NeoBioLab (Cambridge, MA) at >95% purity. L2-N was biotinylated at its C-terminus with the N-terminus unmodified, while all the other peptides were biotinylated at their N-terminus and amidated at their C-terminus. L2-N was dissolved in sterile deionized water containing 0.01% sodium azide, L2-M was initially solubilized in a small amount of DMSO (~ 70–80 μl) and then dissolved in sterile deionized water with 0.01% sodium azide. L2-C was initially resuspended in 30% acetic acid and DMSO (~ 70 μl each), and then dissolved in sterile deionized water with 0.01% sodium azide. The FYL, YYML, DM and WLM peptides were initially solubilized in a small amount of 30% acetic acid (~80 μl), and then dissolved in sterile deionized water with 0.01% sodium azide. Peptide stocks (3.5–5.6 mg/ml) were aliquoted and stored at-20°C.

HeLa or HaCaT cells plated in six-well plates were lysed at ~80% confluency with 500 μl RIPA-MOPS buffer (20mM morpholinepropanesulfonic acid [pH 7.0], 150mM NaCl, 1% Nonidet P-40, 1mM EDTA, 1% deoxycholic acid, 0.1% sodium dodecyl sulfate [SDS]) supplemented with protease inhibitors (1X HALT protease and phosphatase inhibitor cocktail [Thermo Scientific]) or with 500 μl HEPES buffer (20mM Hepes pH8, 50mM NaCl, 5mM MgCl_2_, 1mM dithiothreitol (DTT), and 1.0% triton X-100) containing 1:100 Halt TM Protease & Phosphatase Inhibitors (Thermo Scientific, Prod # 78443). The lysate was centrifuged at 14,000 rpm for 20 min, and the supernatant was incubated with 10 μg of a biotinylated peptide for two hours at 4°C. 40 μl of streptavidin agarose beads slurry (Pierce, cat# 20349) was added, and the mixture was gently rocked for 45 min at 4°C. Beads were recovered by centrifugation and washed four times with RIPA-MOPS buffer supplemented with NaCl to a total of 0.4M or with HEPES buffer. Samples were analyzed by SDS-PAGE and immunoblotting with Vps35 (Abcam, ab57632) or Vps29 (Santa Cruz, sc-98611) antibody.

### Maltose binding protein fusion protein experiments

Individual human Vps26, Vps29, and GST-tagged Vps35 subunits were expressed individually in *E*. *coli*, and the assembled trimeric retromer complex was immobilized on GSH resin via the GST-tag on Vps35 as described [45]. Maltose binding protein (MBP)-L2–6His fusion proteins containing a C-terminal segment from wild-type or mutant HPV16 L2 (amino acids 434–457) were expressed in bacteria and purified using the AKTA-Prime plus FPLC system equipped with a His-trap column. The sequence appended to the C-terminus of MBP in the pMal-C2 expression vector (New England Biolabs) was GSASPQYTIIADAGDFYLHPSYYMLRKHHHHHHC (L2 sequence underlined). Purified proteins were exchanged into 20mM Hepes pH 8, 50mM NaCl and quantified by bicinchoninic acid assay. Five or 10 μM of each fusion protein was incubated with assembled retromer trimer immobilized on GSH resin for two hours at 4°C in 20mM HEPES pH 8.0, 50mM NaCl, 5mM MgCl_2_, 1mM DTT, and 0.1% Triton X-100. Beads were washed twice in HEPES buffer, suspended in SDS loading buffer, boiled, and subjected to SDS-PAGE and anti-His immunoblotting. Bands corresponding to the MBP-L2-His constructs were quantified by Image Lab.

## Supporting Information

S1 Fig L2 mutations do not affect PsV morphology or assembly. **A**. Optiprep gradient-purified, FLAG-tagged wild-type HPV16 or HPV16.L2DM PsV were adsorbed on carbon coated copper grids and stained with Nano-W. The samples were visualized by transmission electron microscopy. **B. and C**. SDS-polyacrylamide gel electrophoresis, staining, and immunoblotting were used to assess relative levels of L1 and L2 in gradient-purified PsV containing FLAG-tagged wild-type L2 or the indicated mutant L2. PsVs containing the same number of encapsidated genomes were denatured in SDS and DTT, loaded in each lane, and after electrophoresis, subjected to silver staining **(B)** or immunoblotting **(C)** to detect L1 and FLAG-tagged L2.
(TIF)

S2 Fig Retromer knock-down inhibits infection of HaCaT cells. HaCaT cells were transfected with an siRNA targeting a retromer subunit, Vsp26, Vsp29, or Vps35. Twenty-four hours later, cells were infected with HPV16 PsV at MOI ~0.5 GFP-transducing particles per cell. Forty-eight hours after infection, successful infection was assessed by flow cytometry for GFP. Results are expressed relative to cells transfected with RISC-free siRNA (set at 100%).
(TIF)

S3 Fig Retromer knock-down is effective. HeLa cells were reverse transfected with the following siRNAs: lane 1, RISC-free; lane 2, si-Vps26 #1; lane 3, si-Vps26 #2; lane 4, si-Vps29; lane 5, si-Vps35 #1; lane 6, si-Vps35 #2. Forty-eight hrs after transfection, extracts were prepared and analyzed by immunoblotting for the level of Vps35. Knock-down of any subunit lowered the amount of Vps35 because the stability of the complex requires all three subunits.
(TIF)

S4 Fig FYL can substitute for a retromer sorting signal. The figure shows the component images used to generate Fig. 2B. Each row shows the same field.
(TIF)

S5 Fig L2 double mutation does not interfere with internalization or capsid disassembly. HeLa cells were mock-infected or infected with FLAG-tagged HPV16 at MOI of 20 or HPV16.L2DM (containing an equivalent number of encapsidated reporter plasmids). Eight hours post-infection, cells were fixed, permeabilized, and stained with anti-FLAG (top panel) or 33L1–7 antibody (bottom panel) (both in red). Nuclei were stained blue with DAPI. A single plane in the Z-dimension is shown in each panel.
(TIF)

S6 Fig Retromer action is required for endosome exit in HaCaT cells. **A**. HaCaT cells were infected and analyzed by PLA as described in the legend to Fig. 3. **B**. Results from the experiment shown in panel A were quantified as described in Fig. 4. Similar results were obtained in two independent experiments.
(TIF)

S7 Fig L2-C peptide binds to Vps35 and Vps29. Wild-type L2-C peptide (WT) or a peptide containing mutations in both retromer binding sites (DM) were incubated with HeLa cell lysate. After streptavidin pull-down and SDS-polyacrylamide gel electrophoresis, bound Vps35 and Vps29 were detected by immunoblotting.
(TIF)

S8 Fig YYML mutation does not totally disrupt retromer binding. Experiment performed as in Fig. 6B, left panel, showing weak binding of retromer by the YYML mutant peptide (which contains an intact FYL site).
(TIF)

S1 Table This table lists oligonucleotides used for site-directed mutagenesis to introduce mutations into the C-terminus of the HPV16 L2 protein, oligonucleotides used as qPCR primers to quantify incorporation of reporter plasmids into pseudoviruses, and siRNAs used for knock-down of mRNAs encoding retromer subunits. The Dharmacon catalogue numbers are shown for the siRNAs.
(DOCX)
