# Supplementary figures and images for: Direct Binding of Retromer to Human Papillomavirus Type 16 Minor Capsid Protein L2 Mediates Endosome Exit during Viral Infection
Source: PLoS Pathog. 2015 Feb 18;11(2):e1004699. doi: 10.1371/journal.ppat.1004699 (PMC4334968; doi:10.1371/journal.ppat.1004699)

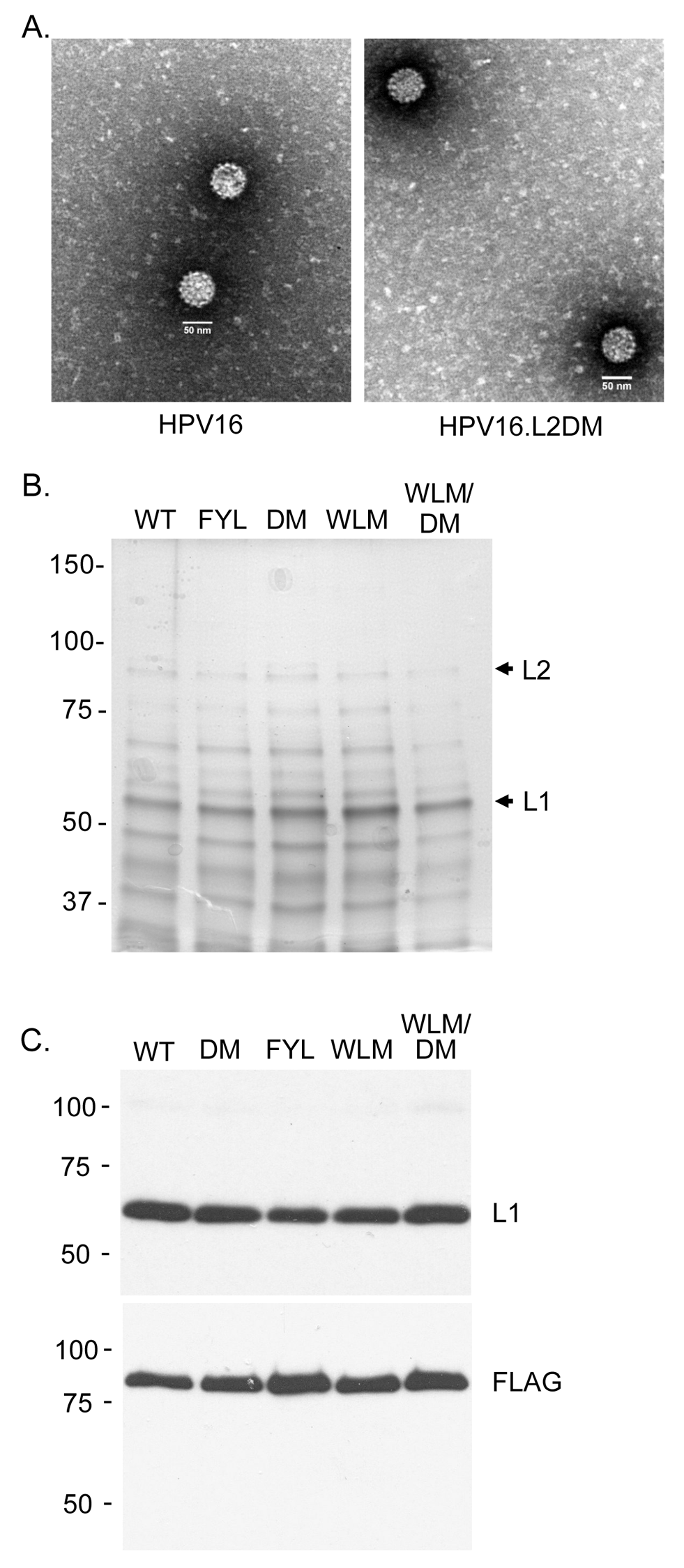

Supplement: S1 Fig — A. Optiprep gradient-purified, FLAG-tagged wild-type HPV16 or HPV16.L2DM PsV were adsorbed on carbon coated copper grids and stained with Nano-W. The samples were visualized by transmission electron microscopy. B. and C. SDS-polyacrylamide gel electrophoresis, staining, and immunoblotting were used to assess relative levels of L1 and L2 in gradient-purified PsV containing FLAG-tagged wild-type L2 or the indicated mutant L2. PsVs containing the same number of encapsidated genomes were denatured in SDS and DTT, loaded in each lane, and after electrophoresis, subjected to silver staining (B) or immunoblotting (C) to detect L1 and FLAG-tagged L2. (TIF) [file ppat.1004699.s001.tif]

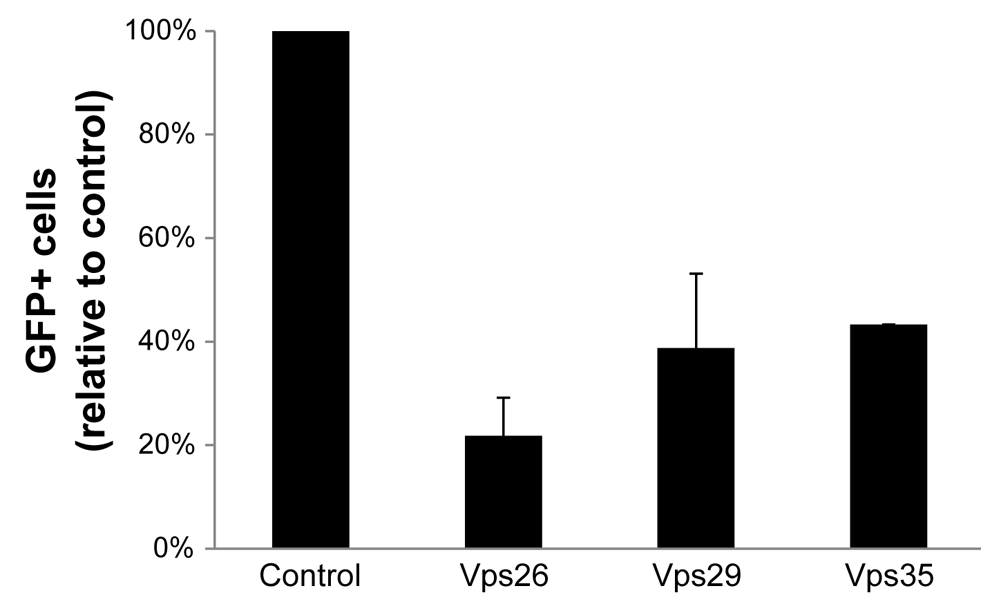

Supplement: S2 Fig — HaCaT cells were transfected with an siRNA targeting a retromer subunit, Vsp26, Vsp29, or Vps35. Twenty-four hours later, cells were infected with HPV16 PsV at MOI ~0.5 GFP-transducing particles per cell. Forty-eight hours after infection, successful infection was assessed by flow cytometry for GFP. Results are expressed relative to cells transfected with RISC-free siRNA (set at 100%). (TIF) [file ppat.1004699.s002.tif]

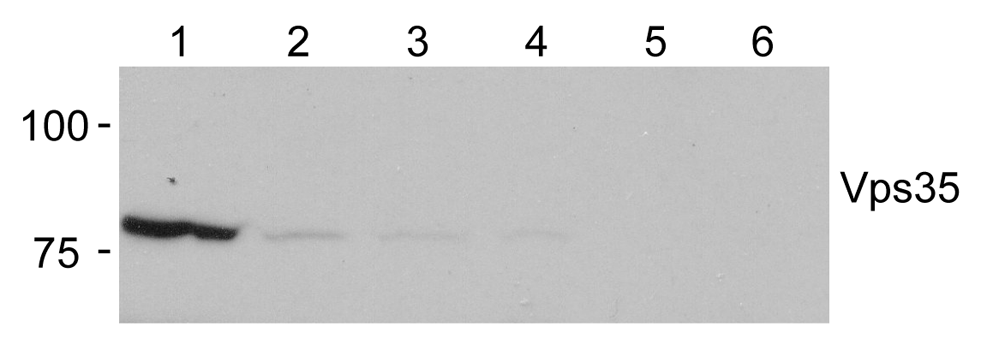

Supplement: S3 Fig — HeLa cells were reverse transfected with the following siRNAs: lane 1, RISC-free; lane 2, si-Vps26 #1; lane 3, si-Vps26 #2; lane 4, si-Vps29; lane 5, si-Vps35 #1; lane 6, si-Vps35 #2. Forty-eight hrs after transfection, extracts were prepared and analyzed by immunoblotting for the level of Vps35. Knock-down of any subunit lowered the amount of Vps35 because the stability of the complex requires all three subunits. (TIF) [file ppat.1004699.s003.tif]

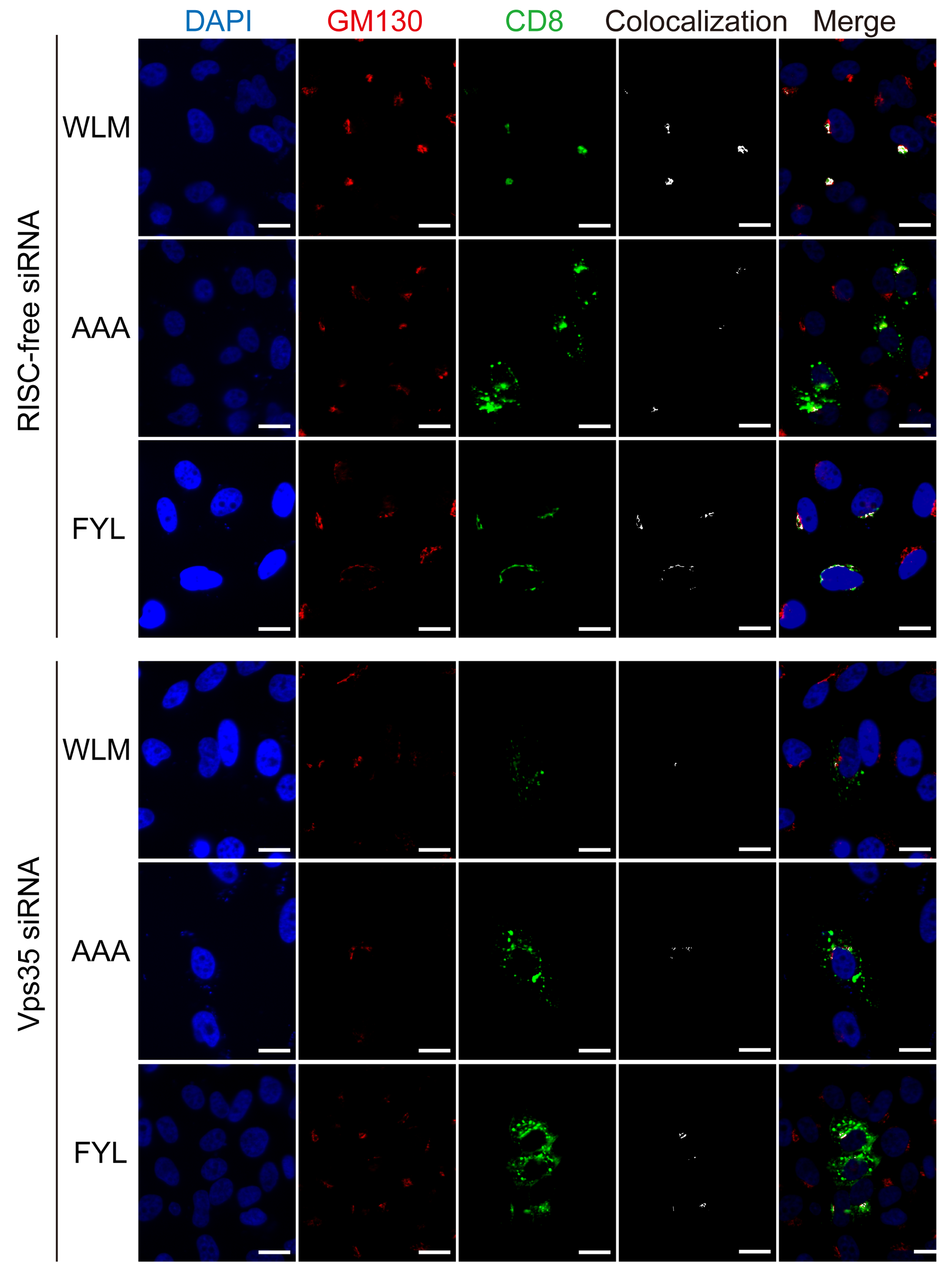

Supplement: S4 Fig — The figure shows the component images used to generate Fig. 2B. Each row shows the same field. (TIF) [file ppat.1004699.s004.tif]

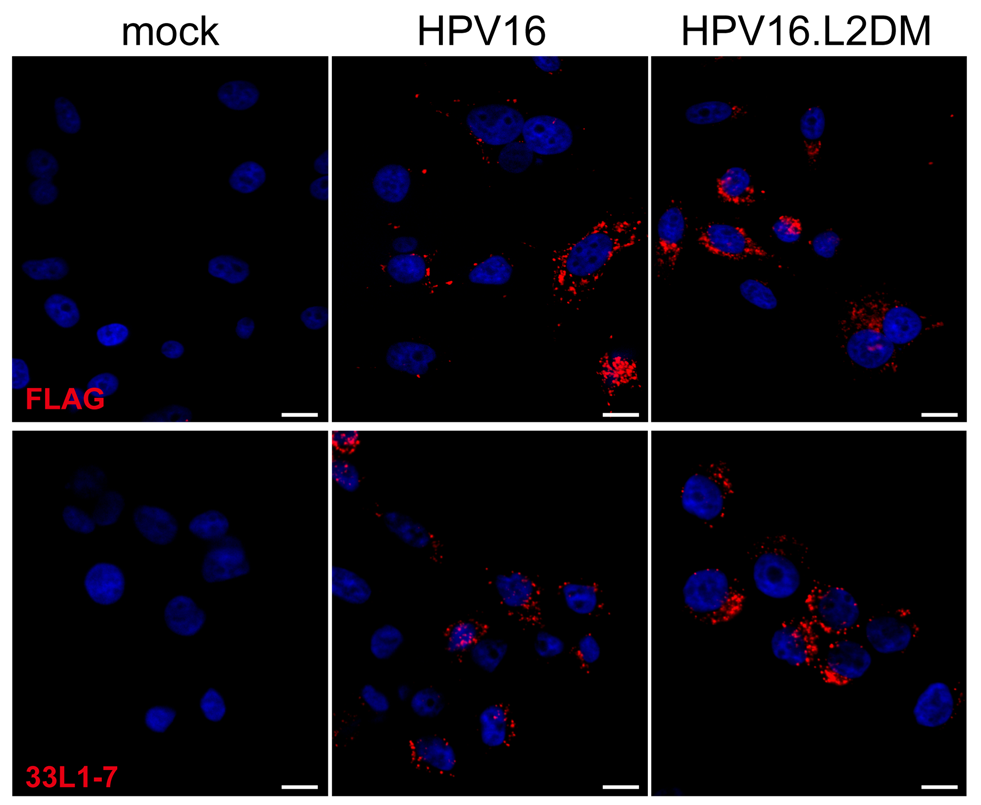

Supplement: S5 Fig — HeLa cells were mock-infected or infected with FLAG-tagged HPV16 at MOI of 20 or HPV16.L2DM (containing an equivalent number of encapsidated reporter plasmids). Eight hours post-infection, cells were fixed, permeabilized, and stained with anti-FLAG (top panel) or 33L1–7 antibody (bottom panel) (both in red). Nuclei were stained blue with DAPI. A single plane in the Z-dimension is shown in each panel. (TIF) [file ppat.1004699.s005.tif]

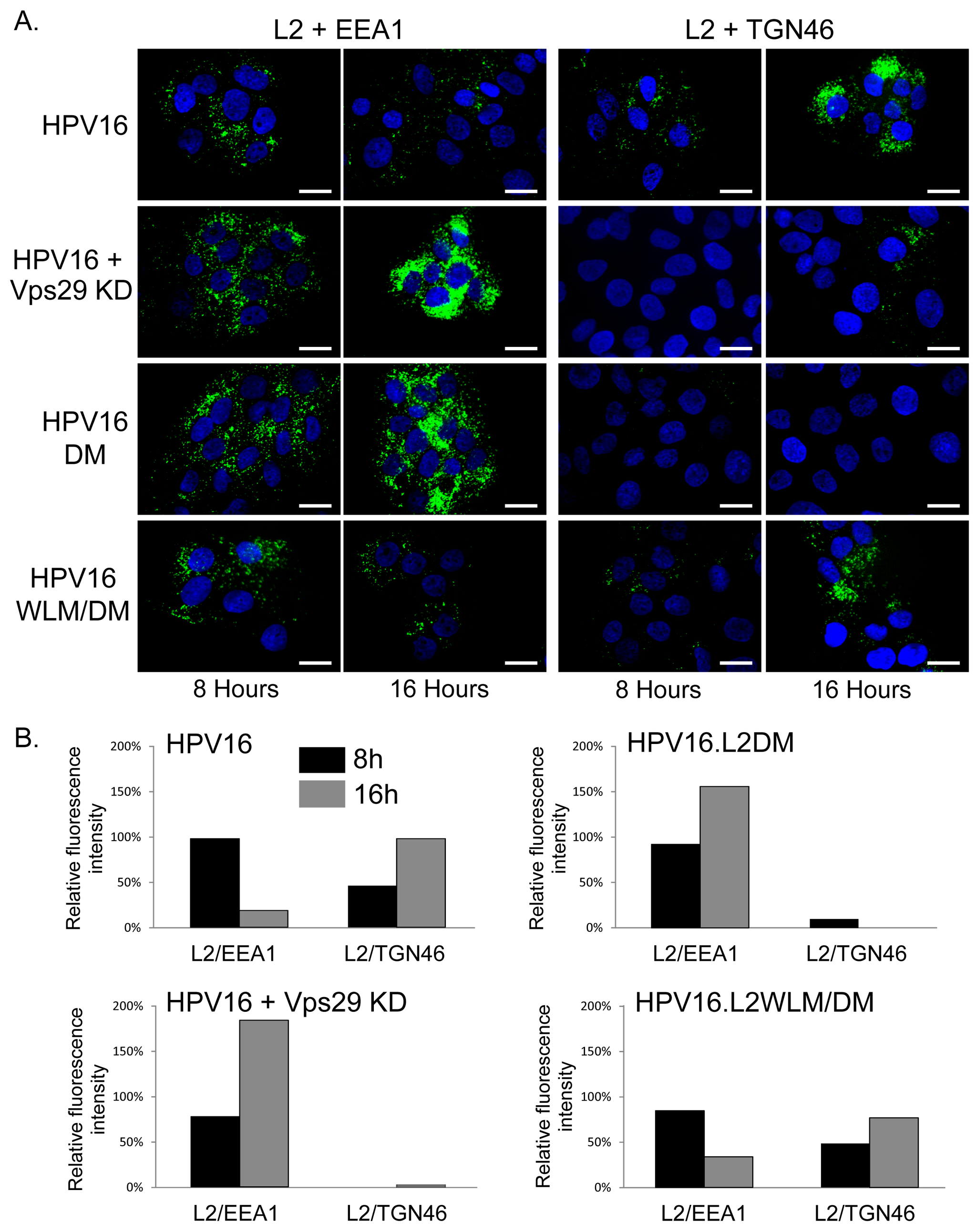

Supplement: S6 Fig — A. HaCaT cells were infected and analyzed by PLA as described in the legend to Fig. 3. B. Results from the experiment shown in panel A were quantified as described in Fig. 4. Similar results were obtained in two independent experiments. (TIF) [file ppat.1004699.s006.tif]

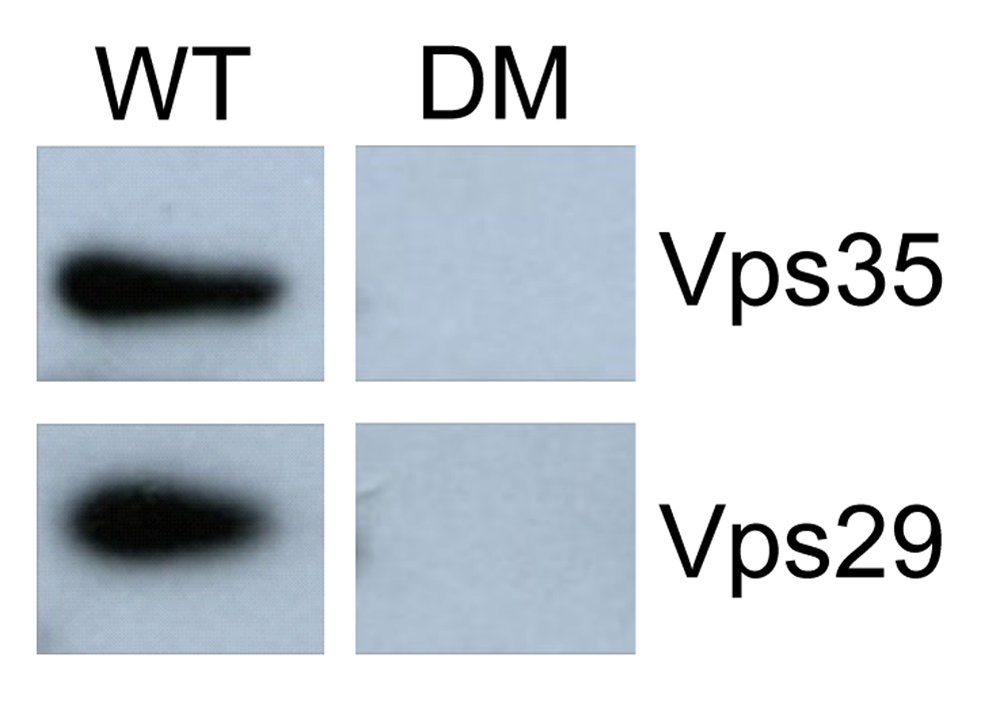

Supplement: S7 Fig — Wild-type L2-C peptide (WT) or a peptide containing mutations in both retromer binding sites (DM) were incubated with HeLa cell lysate. After streptavidin pull-down and SDS-polyacrylamide gel electrophoresis, bound Vps35 and Vps29 were detected by immunoblotting. (TIF) [file ppat.1004699.s007.tif]

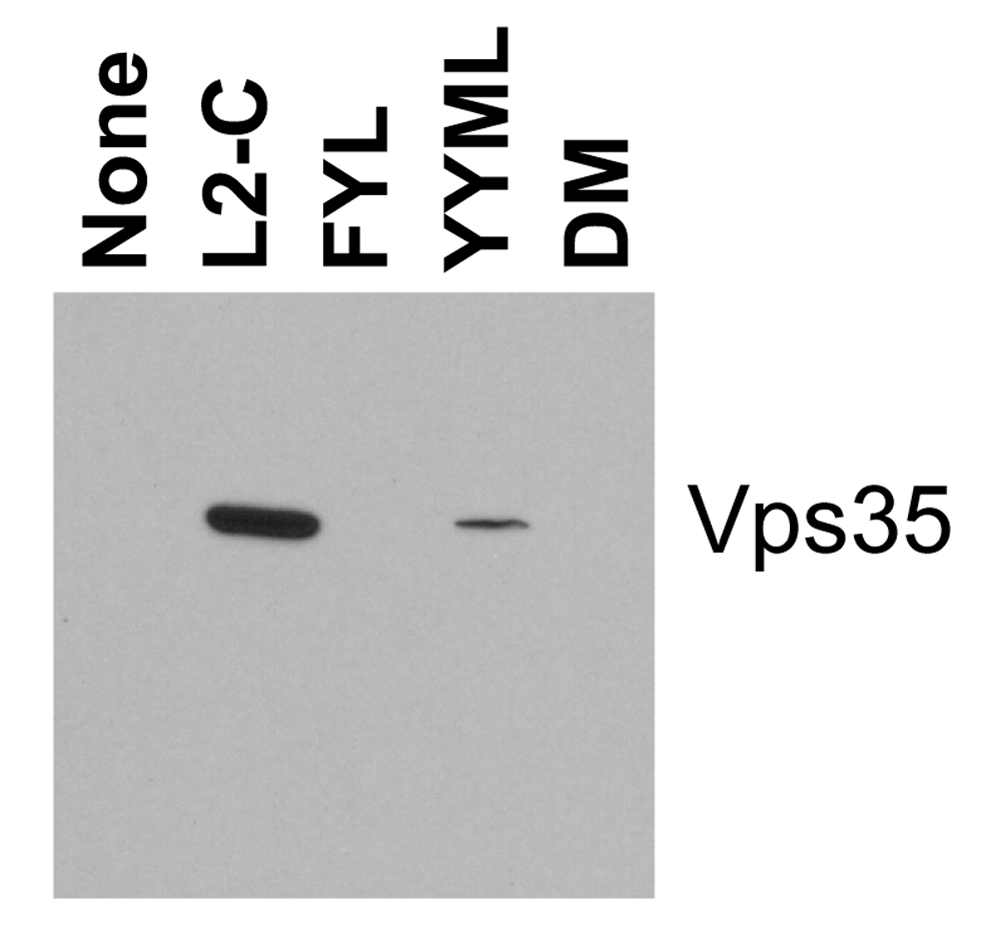

Supplement: S8 Fig — Experiment performed as in Fig. 6B, left panel, showing weak binding of retromer by the YYML mutant peptide (which contains an intact FYL site). (TIF) [file ppat.1004699.s008.tif]
