# Supplementary material for: Direct Binding of Retromer to Human Papillomavirus Type 16 Minor Capsid Protein L2 Mediates Endosome Exit during Viral Infection
Source: PLoS Pathog. 2015 Feb 18;11(2):e1004699. doi: 10.1371/journal.ppat.1004699 (PMC4334968; doi:10.1371/journal.ppat.1004699)
Supplement: S1 Table — The Dharmacon catalogue numbers are shown for the siRNAs. (DOCX) [file ppat.1004699.s009.docx]

**Supporting InformationTable S1.**

Primers used for site-directed mutagenesis of the C-terminus of HPV16.L2

| 1 | YTII to AAAA - Fwd | CGTGCCCGGCAGCCCCCAGGCCGCCGCCGCCGCCGACGCCGGCGACTTCTAC |
| --- | --- | --- |
| 2 | YTII to AAAA - Rev | GTAGAAGTCGCCGGCGTCGGCGGCGGCGGCGGCCTGGGGGCTGCCGGGCACG |
| 3 | DAGD to AAAA - Fwd | CAGTACACCATCATCGCCGCCGCCGCCGCCTTCTACCTGCACCCCAGCTAC |
| 4 | DAGD to AAAA -Rev | GTAGCTGGGGTGCAGGTAGAAGGCGGCGGCGGCGGCGATGATGGTGTACTG |
| 5 | FYL to AAA - Fwd | CATCGCCGACGCCGGCGACGCCGCCGCGCACCCCAGCTACTACATG |
| 6 | FYL to AAA - Rev | CATGTAGTAGCTGGGGTGCGCGGCGGCGTCGCCGGCGTCGGCGATG |
| 7 | HPS to AAA - Fwd | CGCCGACGCCGGCGACTTCTACCTGGCCGCCGCCTACTACATGCTGAGGAAGAGG |
| 8 | HPS to AAA - Rev | CCTCTTCCTCAGCATGTAGTAGGCGGCGGCCAGGTAGAAGTCGCCGGCGTCGGCG |
| 9 | YYML to YAAA - Fwd | CCTGCACCCCAGCTACGCCGCGGCGAGGAAGAGGAGGAAGAG |
| 10 | YYML to YAAA - Rev | CTCTTCCTCCTCTTCCTCGCCGCGGCGTAGCTGGGGTGCAGG |
| 11 | YYML to AAAA - Fwd | CCTGCACCCCAGCGCTGCCGCGGCGAGGAAGAGGAGGAAGAG |
| 12 | YYML to AAAA - Rev | CTCTTCCTCCTCTTCCTCGCCGCGGCAGCGCTGGGGTGCAGG |
| 13 | FYL to WLM - Fwd | CACCATCATCGCCGACGCCGGCGACTGGCTCATGCACCCCAGCTACTACATGCTG |
| 14 | FYL to WLM - Rev | CAGCATGTAGTAGCTGGGGTGCATGAGCCAGTCGCCGGCGTCGGCGATGATGGTG |

GFP or Far-red primers used for qPCR

| GFP - forward | ACGTAAACGGCCACAAGTTC |
| --- | --- |
| GFP - reverse | AAGTCGTGCTGCTTCATGTG |
| Far red forward | GCACCCAGAGCATGAGAAT |
| Far red reverse | TCGTAGGTGGTGGTTCTCT |

siRNA used for retromer subunit knock-down

| VPS26-1 | CCACCUAUCCUGAUGUUAA | D-013195-01 |
| --- | --- | --- |
| VPS26-2 | GCUAGAACACCAAGGAAUU | D-013195-03 |
| VPS29 | UCGAUGAGAAUCUGAAUUA | D-009764-03 |
| VPS35-1 | UUACAAGUCUCUCUGAUUA | D-010894-01 |
| VPS35-2 | GAACAUAUUGCUACCAGUA | D-010894-02 |
